# Microbial transformations of selenite by methane-oxidizing bacteria

**DOI:** 10.1007/s00253-017-8380-8

**Published:** 2017-06-23

**Authors:** Abdurrahman S. Eswayah, Thomas J. Smith, Andreas C. Scheinost, Nicole Hondow, Philip H. E. Gardiner

**Affiliations:** 1 Biomolecular Sciences Research Centre, Sheffield Hallam University, Sheffield, UK; 2 Biotechnology Research Centre, Tripoli, Libya; 3 The Rossendorf Beamline at ESRF, F-38043 Grenoble, France; 4 Institute of Resource Ecology, Helmholtz Zentrum Dresden Rossendorf, D-01328 Dresden, Germany; 5 School of Chemical and Process Engineering, University of Leeds, Leeds, UK

**Keywords:** Methane-oxidizing bacteria, Microbial transformation, Selenite, Elemental selenium, Bioremediation

## Abstract

Methane-oxidizing bacteria are well known for their role in the global methane cycle and their potential for microbial transformation of wide range of hydrocarbon and chlorinated hydrocarbon pollution. Recently, it has also emerged that methane-oxidizing bacteria interact with inorganic pollutants in the environment. Here, we report what we believe to be the first study of the interaction of pure strains of methane-oxidizing bacteria with selenite. Results indicate that the commonly used laboratory model strains of methane-oxidizing bacteria, *Methylococcus capsulatus* (Bath) and *Methylosinus trichosporium* OB3b, are both able to reduce the toxic selenite (SeO_3_^2−^) but not selenate (SeO_4_^2−^) to red spherical nanoparticulate elemental selenium (Se^0^), which was characterized via energy-dispersive X-ray spectroscopy (EDXS), X-ray absorption near-edge structure (XANES) and extended X-ray absorption fine structure (EXAFS). The cultures also produced volatile selenium-containing species, which suggests that both strains may have an additional activity that can transform either Se^0^ or selenite into volatile methylated forms of selenium. Transmission electron microscopy (TEM) measurements and experiments with the cell fractions cytoplasm, cell wall and cell membrane show that the nanoparticles are formed mainly on the cell wall. Collectively, these results are promising for the use of methane-oxidizing bacteria for bioremediation or suggest possible uses in the production of selenium nanoparticles for biotechnology.

**Electronic supplementary material:**

The online version of this article (doi:10.1007/s00253-017-8380-8) contains supplementary material, which is available to authorized users.

## Introduction

Despite its being one of the least abundant elements in the Earth’s crust, selenium is key to a number of critical biochemical reactions, and in addition to which selenium and its compounds have properties that lend themselves to many agricultural, industrial, medicinal and technological applications. Currently, only about 15% of the 2700 t of Se produced annually is recycled (Haug et al. 2007). Minute quantities of Se are essential for normal biological functions in diverse life forms, mainly as selenocysteine, a genetically encoded amino acid incorporated into the active centres of selenoenzymes including glycine reductases, formate dehydrogenases, glutathione peroxidases, iodothyronine deiodinases and thioredoxin reductases, which play key roles in prokaryotic and eukaryotic cells (Stadtman 1991; Heider and Bock 1993; Birringer et al. 2002; Shamberger 2012; Johansson et al. 2005; Patching and Gardiner 1999). However, exposure to excessive amounts of Se released into the environment by human and natural activities can pose serious health risks to humans and may be toxic to other forms of life (Birringer et al. 2002; Lenz and Lens 2009; Qin et al. 2013).

In Se-polluted environments, the element is mainly in the form of the water-soluble and toxic oxyanions, selenite (SeO_3_^2−^) and selenate (SeO_4_^2−^) species. A variety of microorganisms are known to transform the different chemical forms of selenium, and thus play a key role in the recycling of this valuable element and in mitigating its toxicity. Nutritionally and technologically useful forms of selenium can also be produced as a result of such microbial reactions, which include reduction, methylation, oxidation and demethylation. It appears that for most environmental microorganisms, dissimilatory reduction of the Se oxyanions and methylation of the products are the preferred microbial transformation pathways (Dungan and Frankenberger 1999; Eswayah et al. 2016). Dissimilatory reduction of selenite to elemental selenium (Se^0^) has been demonstrated in a wide range of bacteria under aerobic and anaerobic conditions (Switzer Blum et al. 1998; Switzer Blum et al. 2001; Bebien et al. 2001; Klonowska et al. 2005), although the potential contribution of aerobic methane-oxidizing bacteria, which are widespread in the environment, had not until recently been explored.

Aerobic methanotrophs are a diverse and ubiquitous group of bacteria within the environment that are able to grow using methane as their sole source of carbon and energy (Hanson and Hanson 1996; Smith and Murrell 2009). At the centre of biotransformation of organic pollutants by methanotrophs are the methane monoxygenases (MMOs), which naturally oxidize methane to methanol, the first step in the conversion of the former to carbon dioxide and simple one-carbon to four-carbon compounds to form biomass. There are two forms of MMO: soluble (sMMO) found in the cytoplasm and the particulate membrane associated (pMMO). The latter is induced at high copper-to-biomass ratios. It is noteworthy that sMMO and pMMO are different in their protein components and active site metals, but more importantly substrate specificity. Not only can methanotrophs be used to produce useful chemical products on an industrial scale, but in doing so, their ability to use methane, a cheap feedstock but potent greenhouse gas, can be harnessed to reduce its global footprint. The exploitation of methanotrophs in engineered processes has been reviewed by Jiang et al. (2010) and Kalyuzhnaya et al. (2015).

Methanotrophs are capable of remediating a wide range of hydrophobic organic pollutants, and more recently, their capacity for remediating inorganic pollutants has been recognized. Certain methanotrophs have been found able to reduce chromium(VI) to the less toxic and less bioavailable chromium(III) (Al Hasin et al. 2009; Lai et al. 2016b) and to reduce mercuric ions to metallic mercury (Boden and Murrell 2011). Recently, Lai et al. have reported the bioreduction of selenate to elemental selenium using methane as the electron donor in a membrane biofilm reactor containing a microbial community including aerobic methanotrophs (Lai et al. 2016a). These results suggested that methanotrophs can power reduction of selenium species in the environment, but did not establish whether the methanotrophs in the consortium were themselves able to transform selenium species.

In this study, two cultures of the well-characterized pure methanotroph strains, *Methylococcus capsulatus* (Bath) and *Methylosinus trichosporium OB3b*, were chosen for investigation in order to establish whether the pure strains of methane-oxidizing bacteria can biotransform selenium oxyanions. In addition, sMMO-deleted mutant of *Ms. trichosporium* OB3b was used to test the hypothesis that MMO may be involved directly in the reduction of the selenium oxyanions.

## Materials and methods

### Bacterial strains and growth conditions

The methanotrophic bacteria *Mc. capsulatus* (Bath) (NCIBM 11132), *Ms. trichosporium* OB3b (NCIMB 11131) and *Ms. trichosporium* SMDM (a derivative of *Ms. trichosporium* OB3b in which the genes encoding sMMO have been inactivated via marker exchange mutagenesis) (Borodina et al. 2007) were grown and propagated aerobically in sterile nitrate mineral salt (NMS) media (Smith and Murrell 2011) using methane (1:4 *v*/*v* in air) as the source of carbon and energy. The selenium transformation experiments were performed in 50 mL liquid cultures in 250-mL conical Quickfit® flasks capped with Suba-Seals (Sigma-Aldrich) to prevent methane loss while allowing the addition and removal of material using hypodermic syringes. The *Ms. trichosporium* OB3b and *Mc. capsulatus* (Bath) cultures were incubated at the optimum growth temperature of 30 and 45 °C, respectively, on a shaker at 180 rpm and allowed to grow to an OD_600_ of 0.5–0.8. Under the conditions used in these experiments, the *Mc. capsulatus* (Bath) strain grows substantially more quickly than the *Ms. trichosporium* OB3b stain, reaching an OD_600_ of 0.7 typically at 24–30 h, whereas cultures of OB3b take 50–72 h. Addition of either sodium selenite or sodium selenate (Sigma-Aldrich, Dorset, UK) to give the desired selenium concentration was done towards the end of the logarithmic growth phase. The initial selenite concentrations used were 20 and 40 mg L^−1^ for the *Mc. capsulatus* (Bath) and 10 and 20 mg L^−1^ for the *Ms. trichosporium* OB3b stain, respectively. The initial selenate concentration was 10 mg L^−1^ for either strain, respectively. Selenium stock solutions of 1000 mg L^−1^ Se as Na_2_SeO_3_ and Na_2_SeO_4_ were prepared and sterilized by filtration using 0.22-μm syringe filter (Millex®-GP). Three controls were set up for each experiment, with bacterial inoculum, methane and the selenium species omitted, respectively. In order to determine the cellular location of the selenite-reducing activity, grown cultures (an OD_600_ of 0.6–0.7) of both strains were fractionated as previously described by Smith and Foster (1995).

### Investigation of the role of sMMO/pMMO in the reduction selenium oxyanions

In order to investigate the involvement of MMOs (cytoplasmic or sMMO and the membrane-bound or pMMO), *Ms. trichosporium* OB3b cultures at different stages of expression of the MMOs were amended with 10 mg L^−1^ selenite and selenate, respectively. The culture of the sMMO-deleted mutant of *Ms. trichosporium* SMDM was amended with either selenite or selenate and then incubated under the above conditions.

### Quantitation of aqueous selenite and elemental selenium

The selenite and selenate concentrations in the amended cultures were determined by using a HPLC-ICP-MS system. Aliquots (0.5 mL) of the amended cultures were collected at intervals and centrifuged (11,000×*g*; 10 min; room temperature), to remove the cells and other debris. An aliquot of the supernatant (20 μL) was injected by a PerkinElmer LC Flexar autosampler into a PerkinElmer Flexar HPLC pump attached to a Hamilton PRP-X100 column, 4.6 × 250 mm, and coupled to a PerkinElmer ICP-MS NexION 350X. Separation was achieved at a flow rate of 1 mL min^−1^ using a mobile phase made up of 5 mmol L^−1^ ammonium citrate buffer containing methanol (2% *v*/*v*) with the pH adjusted to 5.2.

The pellets were analysed for elemental selenium using a method previously described by Biswas et al. (2011) with minor modifications, as follows. Before analysis, the pellets were washed twice with 1 mL of 1 M NaCl in order to remove non-metabolized selenite. This high concentration of NaCl was employed because it had been previously found to be effective in the collection of colloidal elemental sulphur (Roy and Trudinger 1970). The washed red colloidal selenium was dissolved in 1.5 mL of 1 M Na_2_S, and the solution centrifuged to remove bacterial cells and cell debris.

A standard calibration curve for elemental selenium was constructed using red powdered selenium (Pfaltz & Bauer, Waterbury, USA) dissolved in 1 M Na_2_S solution to give a 1 g L^−1^ stock suspension from which working standards ranging between 10 and 50 mg L^−1^ of elemental selenium were prepared. The absorbance of each of standard solutions and samples were measured at 500 nm.

### Transmission electron microscope and high-angle annular dark-field scanning transmission electron microscopy measurements

Samples of selenite-amended culture (1.5 mL) were pelleted by centrifugation (11,000×*g*; 10 min; room temperature) and washed with 0.1 M sodium phosphate buffer (pH 7.4). The specimens were then fixed in 3% glutaraldehyde in the same buffer overnight at room temperature and washed again in the same buffer. Secondary fixation was carried out in 1% *w*/*v* aqueous osmium tetroxide for 1 h at room temperature followed by the same wash step. Fixed cells were dehydrated through a graded series of ethanol dehydration steps (75, 95 and 100% *v*/*v*) and then placed in a 50/50 (*v*/*v*) mixture of 100% ethanol and 100% hexamethyldisilazane followed by 100% hexamethyldisilazane. The specimens were then allowed to air dry overnight. A small sample of the fixed sample was crushed and dispersed in methanol, with a drop placed on a holey carbon-coated copper grid (Agar Scientific). The samples were examined in an FEI Tecnai F20 field emission gun (FEG)-TEM operating at 200 kV and fitted with a Gatan Orius SC600A CCD camera, an Oxford Instruments X-Max SDD EDX detector and a high-angle annular dark-field (HAADF) scanning TEM (STEM) detector.

### X-ray absorption spectroscopy

For X-ray absorption spectroscopy (XAS) examination, the cultures were grown as described above followed by supplementation with sodium selenite (final concentration of 20 mg L^−1^ Se). After the development of the red colour, the cultures were centrifuged at 11,000×*g* for 10 min. The pellet was freeze dried and analysed without further treatment. Selenium K-edge X-ray absorption near-edge structure (XANES) and extended X-ray absorption fine-structure (EXAFS) spectra were collected at the Rossendorf Beamline at ESRF (Grenoble, France). The energy of the X-ray beam was tuned by a double crystal monochromator operating in channel-cut mode using a Si(111) crystal pair. Two rhodium-coated mirrors were used for collimation and suppression of higher harmonics. A 13-element high-purity germanium detector (Canberra) together with a digital signal processing unit (XIA XMap) was used to measure reaction samples in fluorescence mode. Reference samples were measured in transmission mode using ionization chambers (300 mm, FMB Oxford) filled with 95% N_2_ and 5% Ar (I_0_) and with 100% Ar (I_1_ and I_2_). Spectra were collected at 15 K using a closed cycle He cryostat with a large fluorescence exit window and a low vibration level (CryoVac). Photoinduced redox reactions were effectively prevented by the cold temperature, since XANES edges remained stable during short-term exposure (10 min) as well as during the EXAFS measurements which took up to 8 h. For energy calibration, a gold foil (K-edge at 11,919 eV) was chosen because of its greater inertness in comparison to Se. Data in the XANES region were collected in steps of 0.5 eV, i.e. with higher resolution than the resolution of the Si(111) crystal at the given vertical divergence (1.7 eV) and the broadening due to the core-hole lifetime (2.3 eV). A comparison of single scans of the same sample showed an accuracy of better than 0.5 eV. Dead time correction of the fluorescence signal, energy calibration and the averaging of single scans were performed with the software package SixPack (Webb 2005). Normalization, transformation from energy into k space and subtraction of a spline background was performed with WinXAS using routine procedures (Ressler 1998). The EXAFS data were fit with WinXAS using theoretical backscattering amplitudes and phase shifts calculated with FEFF 8.2 (Ankudinov and Rehr 1997). This method provides a precision of ±0.01 Å for shell distances and a resolution of about ±0.1 Å for neighbouring shells. The error of coordination numbers is ±25%. Statistical analysis of spectra (Eigen analysis and iterative target test) was performed with the ITFA programme package (Rossberg et al. 2003).

### Detection of volatile selenium species

In order to detect volatile selenium compounds, analytical standards of dimethyl selenide (CH_3_SeCH_3_, DMSe) and dimethyl diselenide (CH_3_SeSeCH_3_, DMDSe) (Sigma-Aldrich, Poole, UK, >99.0 and 98%, respectively) were used. Since dimethyl selenenyl sulphide (CH_3_SeSCH_3_, DMSeS) and methylselenol (CH_3_SeH, MeSeH) were commercially unavailable, the compounds were synthesized as described previously (Chasteen 1993) for use as standards. Cultures of *Mc. capsulatus* (Bath) and *Ms. trichosporium* OB3b were grown as detailed above and amended with selenite (40 and 20 mg L^−1^, respectively). Flasks containing medium inoculated with the bacteria but with no SeO_3_^2−^ salts added were run as controls. Samples (200 mL) of the headspace gas were taken through a needle attached to a sorbent tube (Tenax TA/SulfiCarb (C2-CXXX-5314), Markes International, UK) connected to a handheld pump (Easy-VOC grab sampler, Markes International, UK) after 24 and 48 h for *Mc. capsulatus* (Bath) and *Ms. trichosporium* OB3b, respectively. To ensure that the tubes were contamination free, before use, the sorbent tubes were preconditioned with helium at flow rate of 90 mL min^−1^ using the following temperature programme: 15 min at 100 °C, 15 min at 200 °C, 15 min at 300 °C and 15 min at 335 °C.

Analysis of samples was performed on a combined thermal desorption GC-MS system. The volatiles were desorbed at 250 °C and concentrated on a thermal desorber (Unity®, Markes International Limited) at −10 °C cold trap for 5 min (helium flow 50 mL min^−1^) and then were transferred onto the GC/MS system (7890A-GC with 5975C-MS, Agilent Technologies) equipped with a capillary column (Agilent J&W HP-5ms GC Column, 30 m, 0.25 mm, 0.25 μm). Helium was used as the carrier gas at a flow rate of 1 mL min^−1^ and injector temperature of 250 °C, and the chromatogram was obtained using the following temperature programme: 35 °C for 1 min, 10 °C min^−1^ to 250 °C and then held at 250 °C for 1 min. The National Institute of Standards and Technology (NIST) MS search programme (version 2011) was used to identify the compounds based on their mass spectrum.

## Results

### Colour and concentration changes in selenium oxyanion-amended cultures

Each culture medium, after growth to OD_600_ of 0.5–0.8, was amended separately with selenate or selenite in order to test the ability of the two methanotrophic bacteria *Ms. trichosporium* OB3b and *Mc. capsulatus* (Bath) to reduce both selenium oxyanions. Colour changes and the selenate or selenite concentrations in each solution were monitored.

**Fig. 1:**
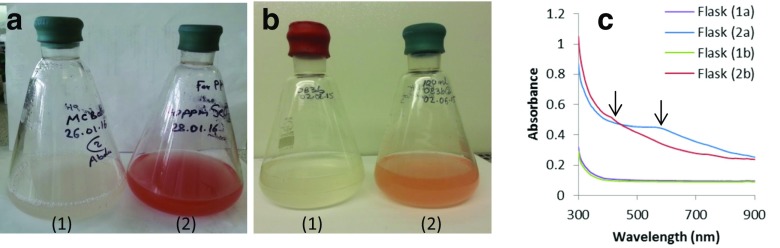
Reduction of SeO_3_^2−^ to red Se^0^ by the methanotrophs *Mc. capsulatus* (**a**) and *Ms. trichosporium* OB3b (**b**) at 48 and 72-h incubation times, respectively. *Left-hand flask* in each image contains no added SeO_3_^2−^; *right-hand flask*, with SeO_3_^2−^ added (40 and 20 mg L^−1^, respectively). **c** Absorption spectra of the contents of the four flasks showing the differences in the absorption peak maximum as reflected in the solution colours

The difference in the colours of the selenite-amended solutions and corresponding spectra of the solutions is shown in Fig. 1. Similar colour change as in the *Ms. trichosporium* OB3b culture medium (see supplementary image S1a) was obtained when *Ms. trichosporium* SMDM (a derivative of *Ms. trichosporium* OB3b in which the genes encoding sMMO have been deleted). Changes in concentration with time at different initial selenite concentrations are shown in S2. Also shown in S1b is evidence that the presence of methane is essential for the reduction of selenite. No colour or concentration changes were observed in the selenate-amended cultures. Results for the determination of the selenate concentrations are shown in supplementary information S3.

### Transformation of selenium oxyanions and elemental selenium content of cultures

**Fig. 2:**
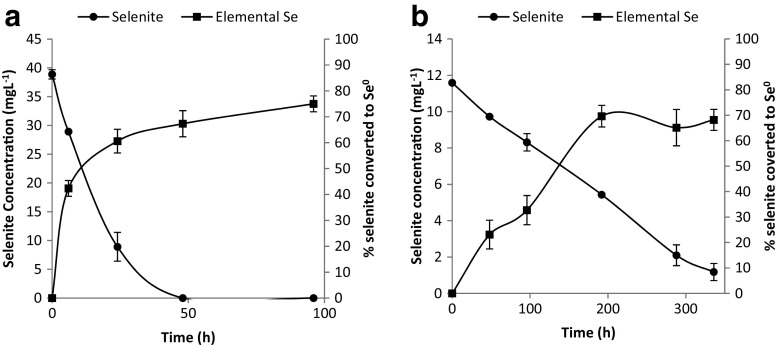
Time course of selenite reduction and % selenite converted to elemental selenium by *Mc. capsulatus* (**a**) and *Ms. trichosporium* OB3b (**b**). Values plotted as mean ± 1 standard deviation (*n* = 3)

Preliminary confirmation that the reddish/yellowish orange suspensions were primarily made up of elemental selenium was obtained by harvesting the particles and subjecting them to sample pretreatment followed by selenium determination using UV-vis spectrometry. As shown in Fig. 2, as the selenite concentrations decreased over time in each culture, the elemental selenium concentrations increased. It is noteworthy that not all the selenite in solution was converted to elemental selenium as shown by the differences in the initial selenite and the final elemental selenium concentrations for both bacteria. In cultures amended with selenate, no change was observed in the selenate concentration during the experiment. This is an indication that neither bacterium is able to reduce selenate to selenium nanoparticles or any other selenium species.

### Time course experiments for the formation of elemental selenium nanoparticles

**Fig. 3:**
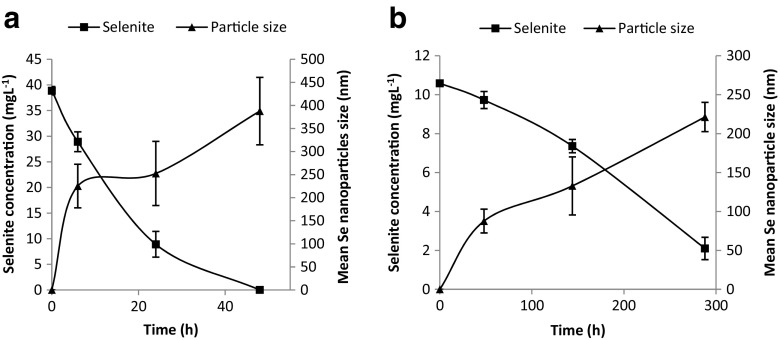
Time course of Se nanosphere growth and SeO_3_^2−^ reduction by *Mc. capsulatus* (**a**) and *Ms. trichosporium* OB3b (**b**)*.* The mean selenium nanoparticle size ± 1 standard deviation (*n* = 140) was measured by TEM

Time course experiments to show the formation of elemental selenium nanoparticles in each selenite-amended culture were performed in order to establish whether the particle sizes changed with incubation time. As shown in Fig. 3, the longer incubation time (see supplementary material S4) for either bacterium resulted in an increase in the mean selenium nanoparticle sizes, obtained by measuring 140 particles at random at the selected time (see supplementary images in S5).

### EXAFS and XANES measurements

**Fig. 4:**
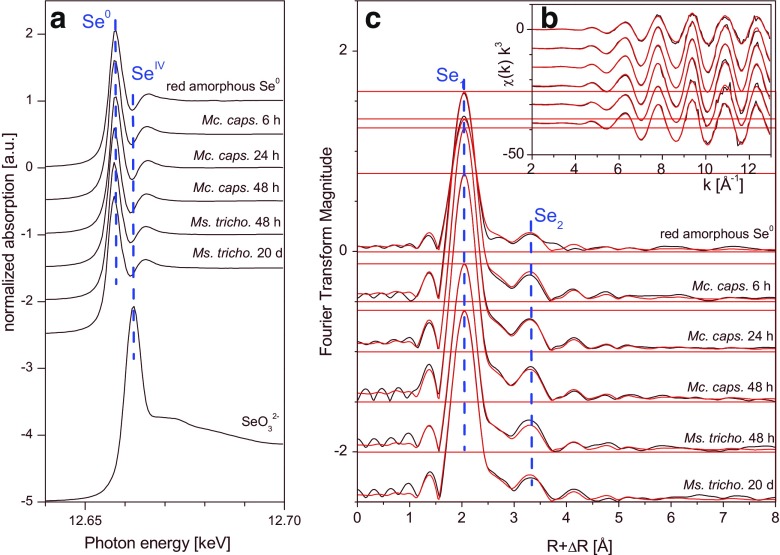
Se K-edge X-ray absorption spectra of cultures of *Mc. capsulatus* and *Ms. trichosporium* and selected references. **a** X-ray absorption near-edge structure (XANES) spectra. **b** Extended X-ray absorption fine structure (EXAFS) spectra. **c** The corresponding Fourier transform magnitude. Experimental data are shown as *black traces*; the *red traces* in **b** and **c** are reconstructions of the experimental data by one principal component

The XANES of all samples shows white line features typical for red Se as shown on top of Fig. 4a. The white line of SeO_3_^2−^ shown at the bottom is about 5 eV higher in energy and coincides with the post-edge minimum of the samples and of red Se^0^, indicating that there are no discernible traces of Se(IV) remaining. The assignment of the spectra as due largely to red elemental selenium is also confirmed by the reconstruction of the EXAFS spectra of all samples by only one principal component, shown as red traces in Fig. 4a, b. The phase identity of red Se^0^ was confirmed by the Fourier transform magnitude (see Fig. 4c), which shows the two Se-Se peaks typical for the crystalline as well as the amorphous variety of red Se. The EXAFS fit shows the typical local structure, with two Se atoms at about 2.35 Å and an additional Se-Se shell at 3.69 Å; the coordination number of this latter shell was much smaller than expected, as has been observed before for amorphous as well as for crystalline red Se (Scheinost and Charlet 2008; Scheinost et al. 2008). The EXAFS fit values also show small variations between the different samples. For strain *Mc. capsulatus*, as well as for *Ms. trichosporium*, the Debye-Waller factors (*σ* ^2^) of both bacteria decrease with reaction time, suggesting an increase of structural order with time (see Table 1) synchronous with the particle growth observed by TEM (see “TEM and HAADF-STEM imaging of cell-associated selenium ” section).

**Table 1:** Se-K edge EXAFS data of Se(IV)-reacted methanotrophs

Sample	CN	*R* (Å)	*σ* (Å^2^)	CN	*R* (Å)	*σ* (Å^2^)	Δ*E*_0_ (eV)	Χres.
*Mc. capsulatus* 6 h	2.2 Se	2.35	0.0030	1.0 Se	3.68	0.0063	10.5	4.2
*Mc. capsulatus* 24 h	2.1 Se	2.35	0.0024	0.9 Se	3.69	0.0047	11.1	2.8
*Mc. capsulatus* 48 h	2.0 Se	2.35	0.0022	0.6 Se	3.69	0.0010	10.8	4.1
*Ms. trichosporium* OB3b 48 h	1.9 Se	2.35	0.0027	0.8 Se	3.69	0.0034	11.0	2.5
*Ms. trichosporium* OB3b 20 day	2.1 Se	2.35	0.0032	0.6 Se	3.70	0.0027	11.5	3.8

### TEM and HAADF-STEM imaging of cell-associated selenium

**Fig. 5:**
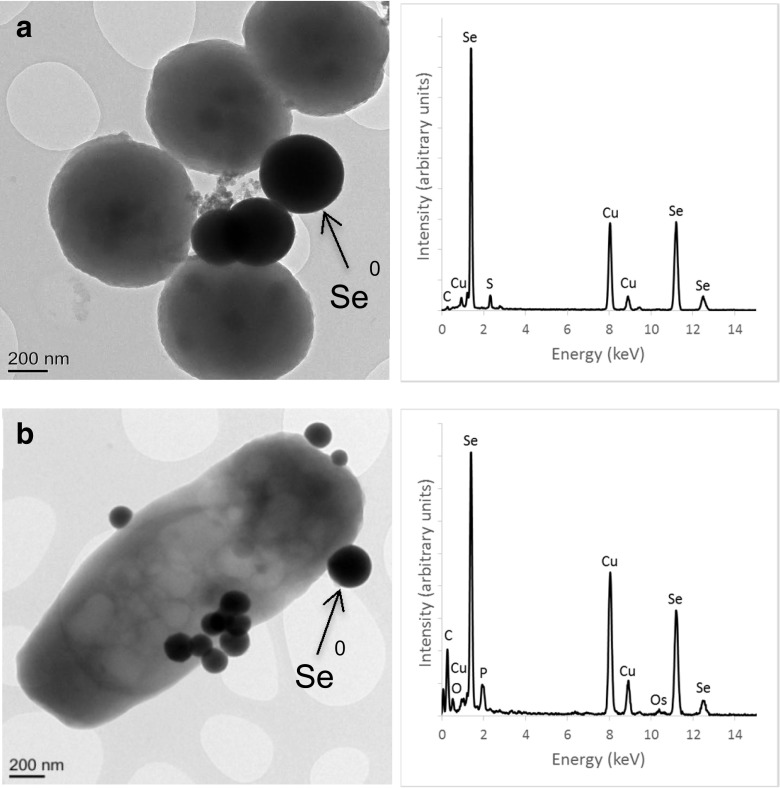
TEM of *Mc. capsulatus* (**a**) and *Ms. trichosporium* OB3b (**b**) cultures exposed to SeO_3_^2−^ (20 mg L^−1^) and EDXS analysis in the electron-dense regions (Se^0^ nanospheres)

Electron micrographs with corresponding EDXS spectra of the cells of the two species of bacteria amended with selenite are shown in Fig. 5. The EDXS analysis of the electron-dense particles shows that they contain selenium, a trace of sulphur and phosphorus in addition to copper from the grid and possibly from the medium and Os from the cell fixing agent. The nanoparticles were spherical and in a variety of sizes. It was found that the mean elemental selenium particle sizes formed in the *Mc. capsulatus* medium were in the main larger than those produced by *Ms. trichosporium* OB3b. This is borne out in the difference in colour intensity of the selenite-amended cultures and confirmation in the differences in the two peak maximum obtained in the spectra of the two solutions. The more intense reddish colour was found in the *Mc. capsulatus* solutions with the larger elemental selenium particles in contrast to the yellowish orange observed in the *Ms. trichosporium* OB3b cultures. The mean particle sizes in the *Mc. capsulatus* cultures was about 387 nm compared to 221 nm for *Ms. trichosporium* OB3b cultures after 48 and 288 h incubation, respectively.

**Fig. 6:**
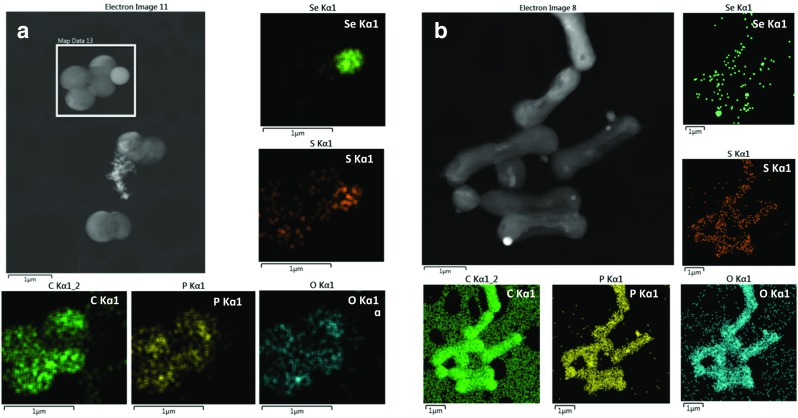
HAADF-STEM imaging of *Mc. capsulatus* (**a**) and *Ms. trichosporium* OB3b (**b**) showing Se nanospheres attached to the cells with EDXS maps (generated from spectra collected from the indicated areas) of relevant elements

The electron micrographs suggest that the elemental selenium nanoparticles are in extracellular space and attached to the surface of the cells suggesting that extracellular selenite reduction is followed by subsequent growth. The HAADF-STEM imaging with EDXS maps of the two bacteria is shown in Fig. 6. The electron micrograph image (white square) of *Mc. capsulatus* (Bath) shows the bacteria and a selenium nanoparticle. The distribution of the elements C, O and P map to the bacteria, and that of Se and S overlap in the area corresponding to the nanoparticle. Similar image and mapping of the same elements for *Ms. trichosporium* OB3b show that C, P and O map well to the bacteria, and S and Se overlap, but this time, the nanoparticles are distributed all over the bacteria.

### Confirmation of the site of selenite reduction and role of methane

In order to confirm the site of selenite reduction and to test the hypothesis that methane gas acts as the source of electrons for the bioreduction of the selenium oxyanions, control experiments were performed with both strains from which methane was omitted, and no red colour was formed in the presence of selenite indicating that the presence of the carbon and energy source methane is needed for reduction of the selenite (see supplementary information S1b). In order to determine the cellular location of the selenite-reducing activity, experiments were performed with cell fractions: cell wall, cell membrane and cytoplasm fractions were separately amended with selenite and monitored visually (see supplementary material S6). The results showed that the red colour of elemental selenium was detected in the cell wall medium, and a weak red tinge in the cell membrane medium probably due to the traces of reductase enzyme(s) contamination, which may have diffused from the cell wall to the cell membrane (Dhanjal and Cameotra 2010).

### Detection and identification of volatile selenium species

**Fig. 7:**
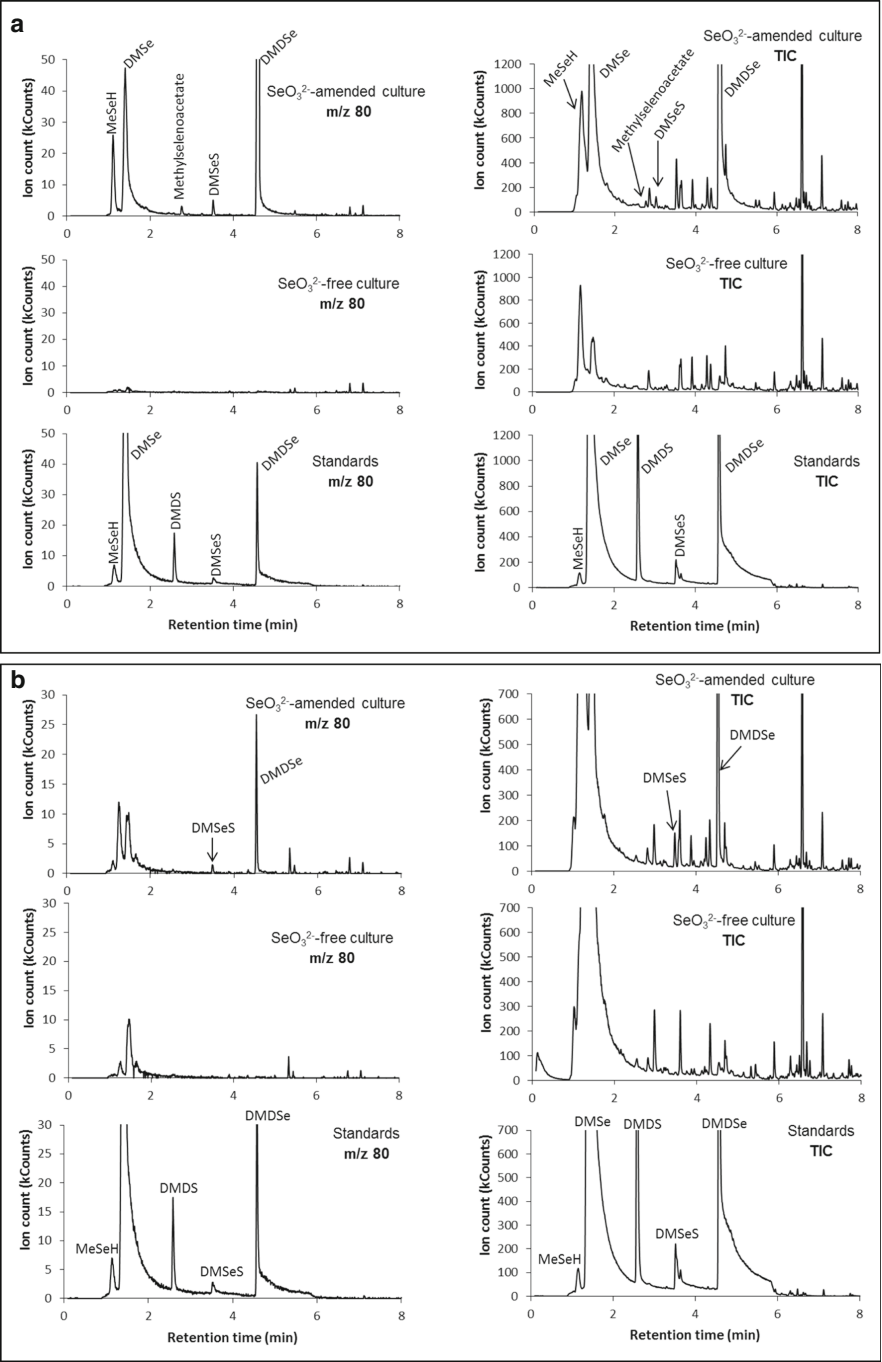
**a** GC-MS chromatograms of the headspace gas of the *Mc. capsulatus* (Bath) cultures amended with (40 mg L^−1^) and without selenite and that of mixed standards containing MSeH, DMSe, dimethyl disulphide (DMDS), DMSeS and DMDSe. **b** GC-MS chromatograms of the headspace gas of the *Ms. trichosporium* OB3b cultures amended with (20 mg L^−1^) and without selenite and that of mixed standards containing MSeH, DMSe, dimethyl disulphide (DMDS), DMSeS and DMDSe

In order to detect the volatile selenium species, the headspace of the incubation flask was sampled using a syringe and injected into a GC-MS after preconcentration of the sample through a sorption tube. It was observed in preliminary experiments with selenite-amended culture medium solutions that the colour of the suspensions tended to fade with time, an indication that Se^0^ was probably being transformed into other selenium species. Indeed, separate experiments with harvested nanoparticles from both bacteria revealed that volatile selenium species were formed in the headspace of the flasks. Interestingly, the distribution profile of the methylated species was different compared to those formed when selenite was added to the culture medium. In the former solutions, three species, dimethyl selenide, dimethyl diselenide and dimethyl selenenyl sulphide, were detected in the headspace of both bacteria. It has been suggested by a number of investigators (Doran and Alexander 1977; Kagami et al. 2013; Chau et al. 1976; McCarthy et al. 1993; Michalke et al. 2000; Chasteen and Bentley 2003) that diverse microbes are capable of transforming selenite into volatile selenium species. In the selenite-amended cultures of either bacterium, it was observed that the volatile selenium species were detected as the red elemental selenium colour was developing. The headspace of the culture medium of both bacteria with and without selenite addition and standards were sampled and analysed for volatile selenium-containing species. GC-MS chromatograms of all the samples analysed are shown in Fig. 7, showing a variety of volatile methylated selenium and mixed selenium-sulphur species produced by both organisms. In addition to the three previously identified methylated species, methyl selenol and methylselenoacetate were detected in the *Mc. capsulatus* (Bath) headspace. In contrast, two selenium species, dimethyl diselenide and dimethyl selenenyl sulphide, were detected. Table 2 presents a summary of the volatile selenium-containing species produced when each bacterium culture is amended with selenite, biogenic selenium produced by the bacterium and commercial amorphous selenium, respectively.

**Table 2:** Volatile selenium species produced by methanotrophs from different selenium-containing substrates

Strain	Substrate	Product				
		DMSe	DMDSe	DMSeS	Methyl selenol	Methylselenoacetate
*Mc. capsulatus*	Selenite	+	+	+	+	+
	Bio-Se^0^	+	+	+	−	−
	Che-Se^0^	+	+	+	−	−
*Ms. trichosporium* OB3b	Selenite	−	+	+	−	−
	Bio-Se^0^	+	+	−	−	−
	Che-Se^0^	+	−	−	−	−

## Discussion

In the selenate-amended cultures, no colour or selenate concentration changes were observed, which is a strong indication that in the presence of either of these two pure strains of methantrophs, selenate is not biotransformed into elemental selenium. This finding is in contrast to that of Lai et al. (2016a), who used a biofilm microbial community in the presence of methane to show that selenate is reduced to elemental selenium. In our experiments, there were changes in the colour and oxyanion concentration only in the selenite-amended solutions. Hence, if the mixed population of methanotrophs in the study of Lai et al. had the same selenium-transforming properties as the pure strains analysed here, the overall reaction to convert selenate to elemental selenium may have been accomplished by the combined activities of methanotrophs and other selenate-reducing, bacteria. Although there were concentration colour changes in both the *Ms. trichosporium* OB3b and *Mc. capsulatus* (Bath) culture media to which selenite was added, the rate at which these occurred was dependant on the type of bacteria used. The slower rate of selenite reduction by the *Ms. trichosporium* OB3b may be linked to its slow growth rate compared with the *Mc. capsulatus* (Bath).

The colour change in the culture medium containing *Mc. capsulatus* was rapid with perceptible reddish tinge occurring in a matter of hours and developing into an intense reddish hue in less than 24 h. In contrast, the colour change in the *Ms. trichosporium* OB3b culture medium was less intense and much slower to develop, appearing after about 2 days. However, it is noteworthy that the nanoparticles may have begun to form long before any perceptible colour change occurs in the solutions as indicated by the reduction in the selenite concentrations at the beginning of the experiments.

The results of the time course experiments (Fig. 3) provide evidence that the initial particles act as nuclei for further growth. In addition, it is indicative that both of these bacteria can be used to produce nanoparticles of a variety of sizes provided that there is timely intervention to stop further nanoparticle growth. TEM images taken at three different times show the growth of the particles (see supplementary information S4). Indeed, in the scheme proposed by Jain et al. (2015), the synthesis of biogenic elemental selenium (BioSeNPs) by an anaerobic granular sludge and wastewater occurs in two steps: initial reduction of selenite to elemental selenium particles either intracellularly or extracellularly followed by growth of the nanoparticles. Intracellularly produced elemental selenium nanoparticles are first coated with protein before they are expelled into extracellular space. Irrespective of the origin of the elemental selenium particles, they are invariably capped and stabilized with extracellular polymeric substances (EPSs) (Jain et al. 2015).

It can be seen from these experiments that for the production of nanoparticles of sizes less than 100 nm, the slower reacting *Ms. trichosporium* OB3b is to be preferred over the faster *Mc. capsulatus.* Further examination of the particles produced by both bacteria for diffraction patterns did not show evidence of any crystalline structure.

A more detailed examination of the particles with the aid of HAADF-STEM imaging and EDXS mapping provided evidence that the maps of selenium and sulphur overlap, which would suggest that both elements are present in a single structure. This is hardly surprising since the initial reactions in the previously proposed pathways for the biological reduction of selenium involve a variety of thiol group-containing compounds, which react as shown in the following equation:

$$ 4\mathrm{RSH}+{\mathrm{H}}_2{\mathrm{SeO}}_3=>\mathrm{RS}-\mathrm{Se}-\mathrm{SR}+\mathrm{RSSR}+3{\mathrm{H}}_2\mathrm{O} $$

The close Se and S mapping within the EDXS images of the cultures would indicate that the pathways involving the reaction of the intermediate RS-Se-SR is likely to result in the co-precipitation of both elements. Of relevance is the observation that both sulphur and selenium are co-precipitated in the presence of sulphate-reducing bacteria (Zannoni et al. 2007). This is further evidence to indicate that there may be reactions common to the biological transformation of both elements. Abiotic and biotic reactions could together account for the co-precipitation of both elements. One example of a possible abiotic reduction reaction involving both selenium and sulphur is given by the following equation:

$$ {{\mathrm{S}\mathrm{e}\mathrm{O}}_3}^{2-}+2{\mathrm{H}\mathrm{S}}^{-}+4{\mathrm{H}}^{+}=>{\mathrm{S}\mathrm{e}}^0+2{\mathrm{S}}^0+3{\mathrm{H}}_2\mathrm{O} $$

proposed by Hockin and Gadd (2003).

The results of the experiments with the cell fractions show that the reduction occurs on the cell wall of both bacteria, which is consistent with the likely extracellular location of the selenium particles that are formed. Reduction of selenite by the cell wall fractions occurred in the absence of methane. Also, since the sMMO-deleted mutant of *Ms. trichosporium* OB3b formed nanoparticles indistinguishable from the wild-type strain, it appears that the components of the sMMO enzyme system (including its NAD(P)H-dependent reductase) are not essential for the reduction of selenite. Since the cell wall fraction of the cells is capable of reducing selenite in the absence of added reducing agents, although the cultures as a whole require methane to perform the reaction, it seems that methane (activated either by sMMO or the particulate methane monooxygenase system) is likely the ultimate source of reducing agents, though other mediator(s) are involved in transferring the electrons to selenite (Smith and Murrell 2011).

Identification of the selenium-containing species in the headspace of the cultures was achieved by matching the retention times of the standards together with mass spectra information stored in the instrument NIST library database. Using this approach, it was possible to detect methyl selenol (CH_3_SeH, MSeH), methylselenoacetate (C_3_H_6_OSe), dimethyl selenide (CH_3_SeCH_3_, DMSe), dimethyl diselenide (CH_3_SeSeCH_3_, DMDSe) and dimethyl selenenyl sulphide (CH_3_SeSCH_3_, DMSeS) in the headspace of selenite-amended *Mc. capsulatus* culture medium. In contrast, only two volatile selenium-containing species, DMDSe and DMSeS, were detected in the headspace of the selenite-amended *Ms. trichosporium* culture. It is noteworthy that with both culture media, these selenium species were detected soon after selenite addition. Results of experiments with the harvested nanoparticles clearly show that these are required for the formation of volatile selenium species, but also, other species are directly formed through other possible pathways as suggested by Challenger (1945) and Reamer and Zoller (1980). The manner in which the methylation of selenium may link to the one-carbon central metabolism of the methanotrophs remains to be established.

The results presented above clearly indicate that the pure strains of the methanotrophic bacteria *Mc. capsulatus* and *Ms. trichosporium* OB3b are able to reduce selenite but not selenate to produce elemental selenium and volatile selenium species. The formation of elemental selenium appears to be mainly an extracellular process, probably accomplished indirectly with electrons derived from methane. It is probable that reducing agents containing sulfhydryl groups on the cell wall plays a key role in the bioreduction process of selenite. This opens the possibility that methanotrophs (which are widespread across diverse environments) may play a significant role in the global selenium cycle. The results also suggest that these bacteria may be useful in preparing selenium nanoparticles of a range of sizes for biotechnological applications. Much remains to be determined about the pathway of selenium biotransformations in methanotrophs, though it appears that elemental selenium may not necessarily be an intermediate on the pathway to the formation of all volatile selenium species.

## Electronic supplementary material

ESM 1 (PDF 683 kb)
