# Supplementary material for: Microbial transformations of selenite by methane-oxidizing bacteria
Source: Appl Microbiol Biotechnol. 2017 Jun 23;101(17):6713–24. doi: 10.1007/s00253-017-8380-8 (PMC5554269; doi:10.1007/s00253-017-8380-8)
Supplement: Supplementary file 1 — (PDF 683 kb) [file 253_2017_8380_MOESM1_ESM.pdf]

Submission ID: AMAB-D-17-00183R2

Applied Microbiology and Biotechnology

Microbial Transformations of Selenite by Methane-Oxidizing Bacteria

Abdurrahman S. Eswayah[a,b], Thomas J. Smith[a], Andreas C. Scheinost[c], Nicole Hondow[d] and Philip H. E. Gardiner[a*]

a. Biomolecular Sciences Research Centre, Sheffield Hallam University, Sheffield, UK

b. Biotechnology Research Centre, Tripoli, Libya

c. The Rossendorf Beamline at ESRF, F-38043 Grenoble, France, and Institute of Resource Ecology, Helmholtz Zentrum Dresden Rossendorf, D-01328 Dresden

d. School of Chemical and Process Engineering, University of Leeds, Leeds, UK

*Corresponding author email address: p.h.gardiner@shu.ac.uk; Telephone: +441142253442; ORCID: 0000-0002-2687-0106.

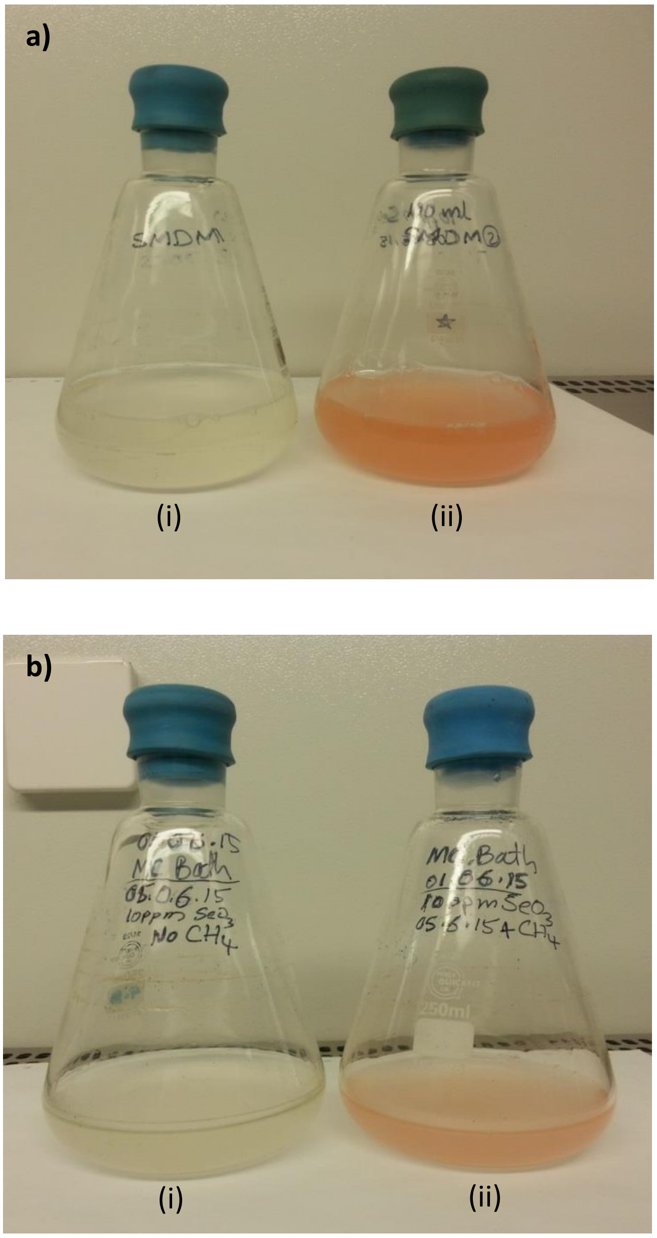

S1

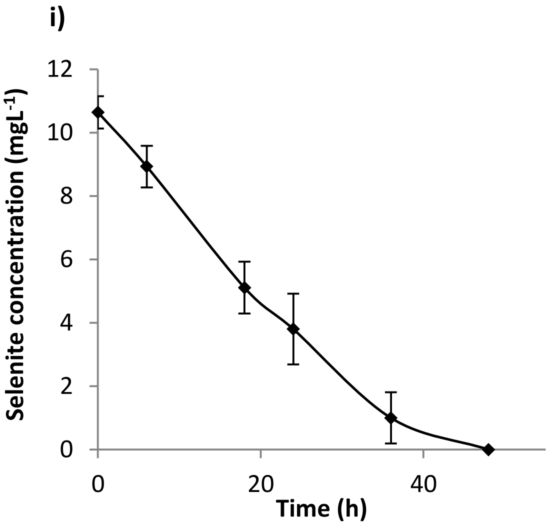
i)
Selenite concentration (mgL-1)
Time (h)
0
20
40
0
2
4
6
8
10
12

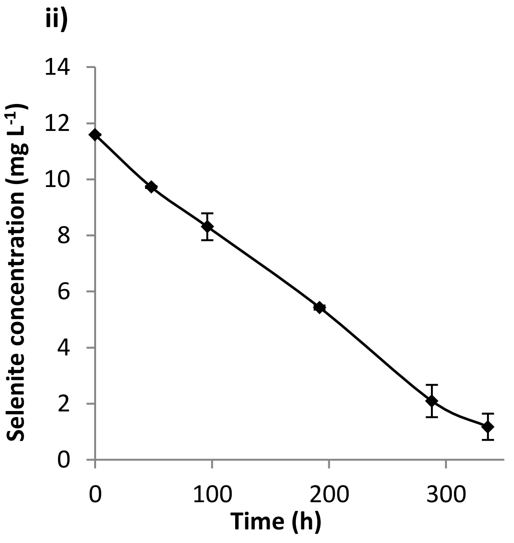
ii)
Selenite concentration (mg L-1)
Time (h)
0
100
200
300
0
2
4
6
8
10
12
14

S2a

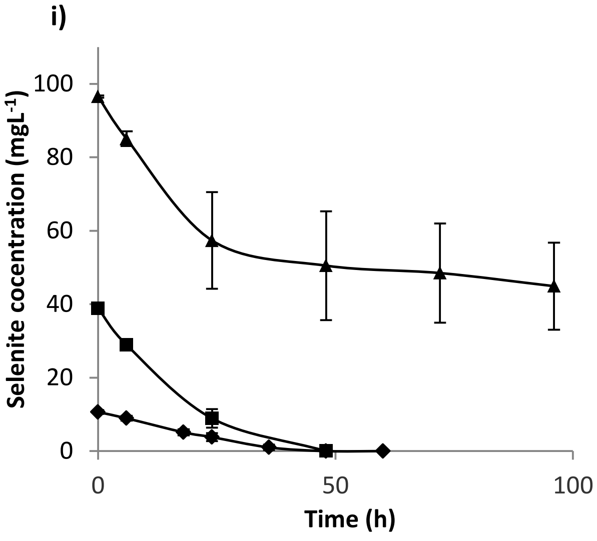
i)
Selenite cocentration (mgL-1)
Time (h)
0
50
100
0
20
40
60
80
100

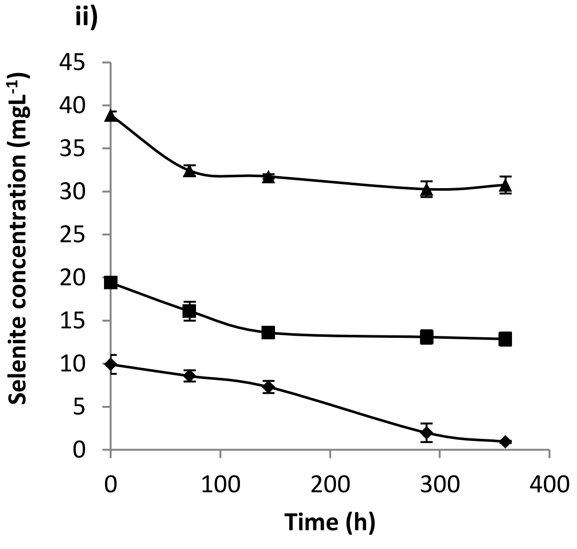
ii)
Selenite concentration (mgL-1)
Time (h)
0
100
200
300
400
0
5
10
15
20
25
30
35
40
45

S2b

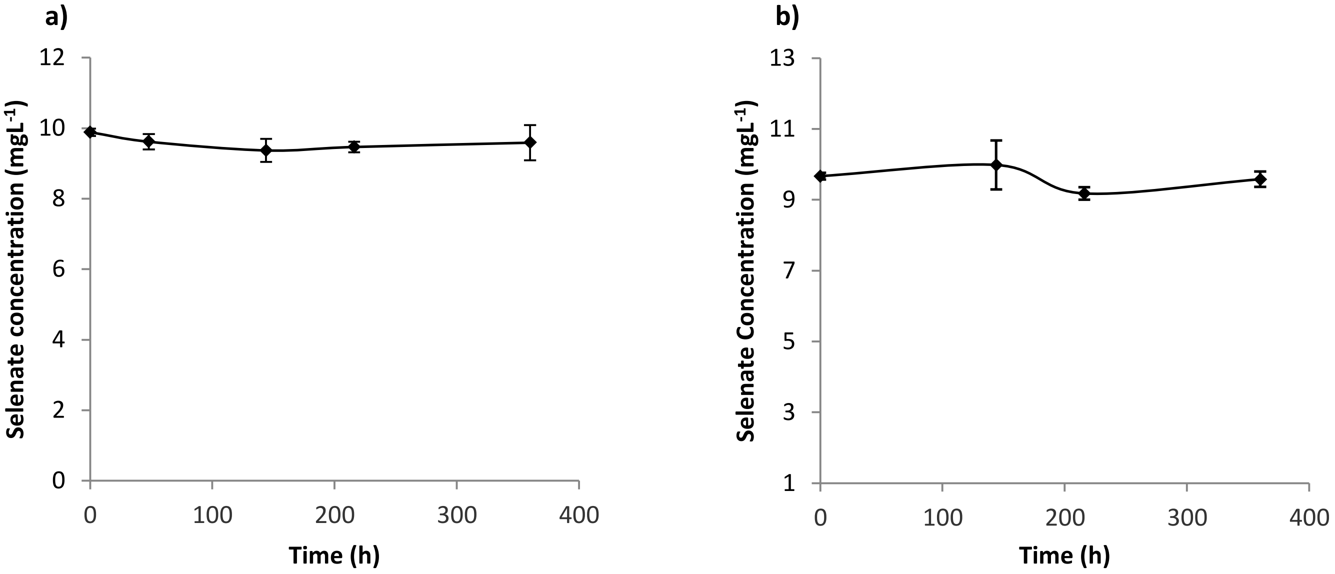
a)
Selenate concentration (mgL-1)
12
10
8
6
4
2
0
0
100
200
300
400
Time (h)
b)
Selenate Concentration (mgL-1)
13
11
9
7
5
3
1
0
100
200
300
400
Time (h)

S3

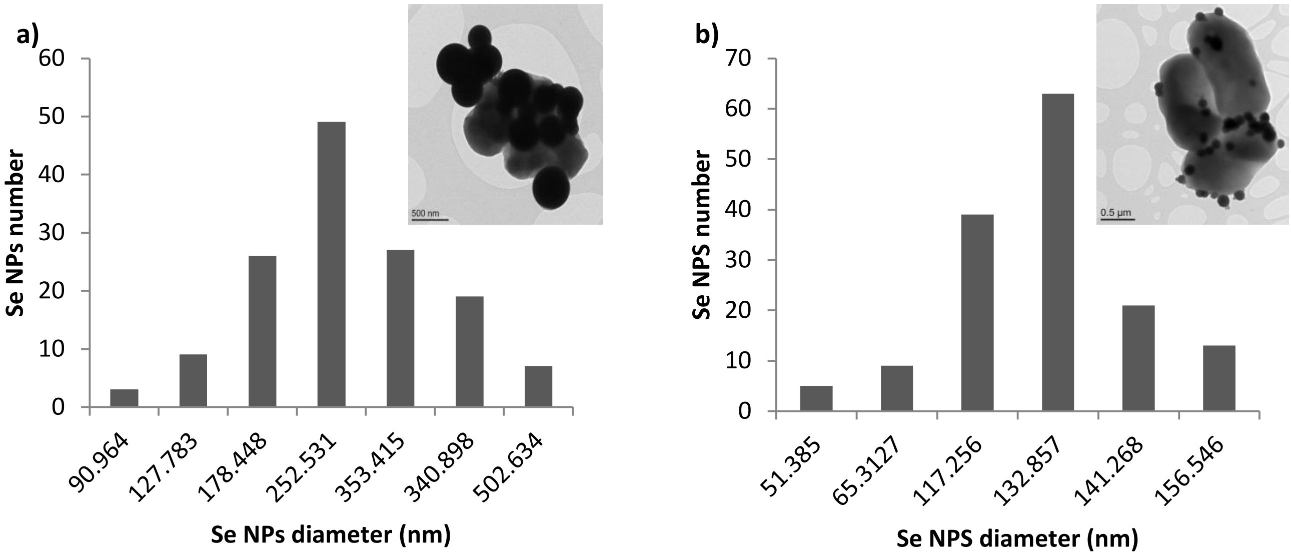
a)
Se NPs number
60
50
40
30
20
10
0
90.964
127.783
178.448
252.531
353.415
340.898
502.634
Se NPs diameter (nm)
500 nm
b)
Se NPS number
70
60
50
40
30
20
10
0
51.385
65.3127
117.256
132.857
141.268
156.546
Se NPS diameter (nm)
0.5 µm

S4

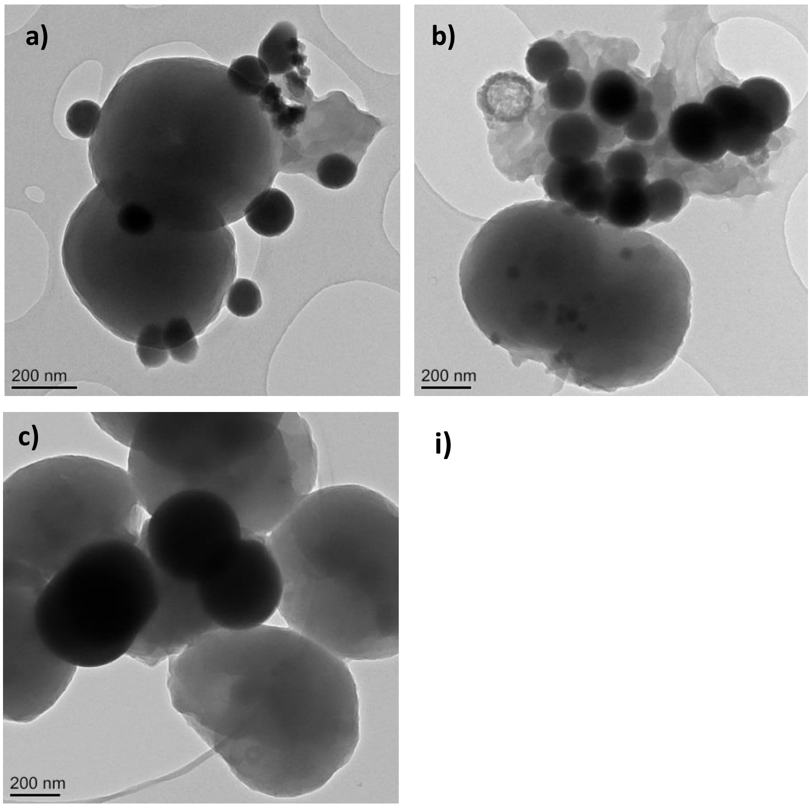

i)

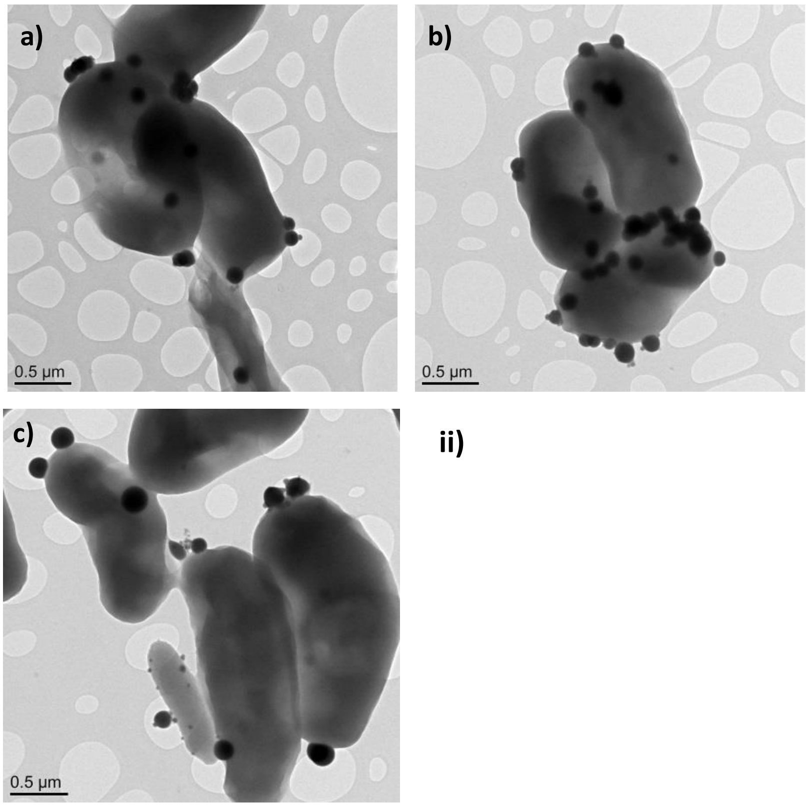

ii)

S5

a)

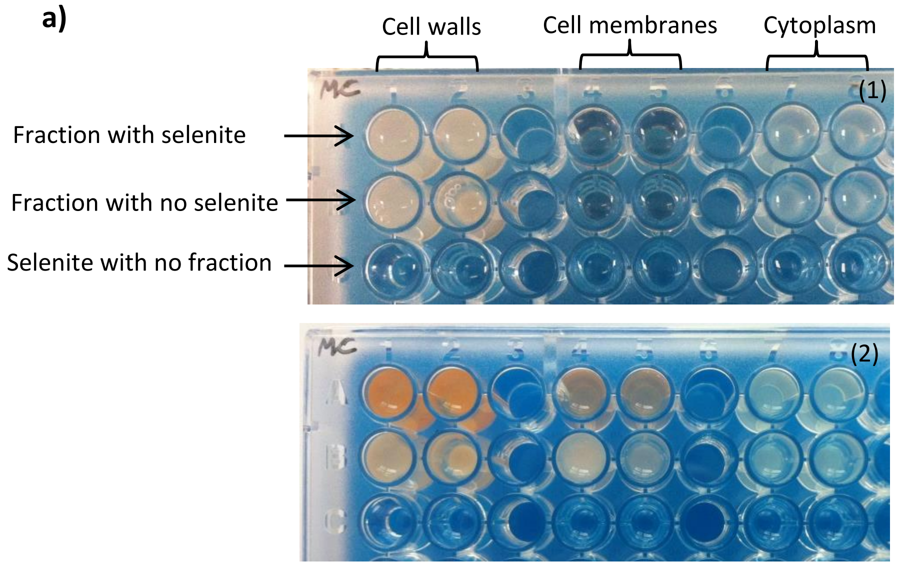
Cell walls
Cell membranes
Cytoplasm
Fraction with selenite
Fraction with no selenite
Selenite with no fraction
(1)
(2)

a)

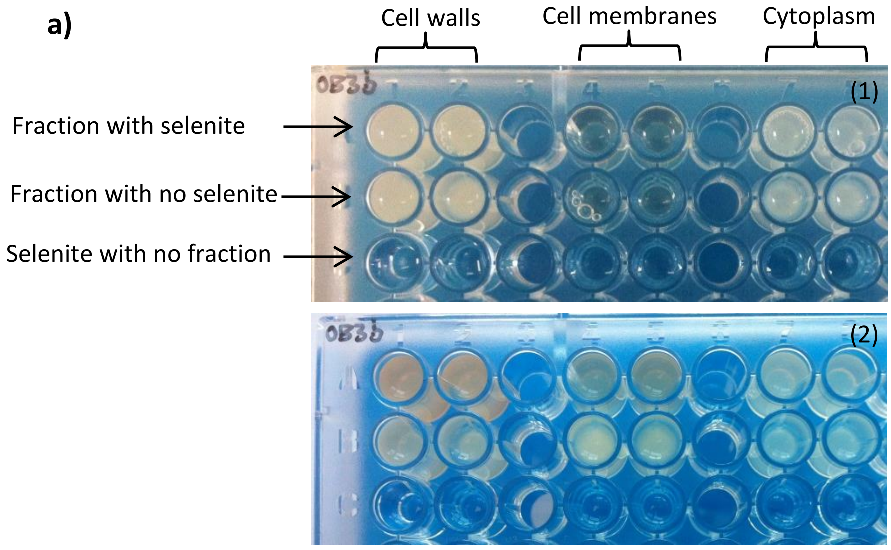
Cell walls
Cell membranes
Cytoplasm
Fraction with selenite
Fraction with no selenite
Selenite with no fraction
(1)
(2)

Legends to Supplementary Figures and Table

S1. Reduction of SeO_3^{2-}(10mg L^{-1}) to red Se^0 by sMMO-deleted mutant of Ms. *trichosporium* OB3b (SMDM) (a) without (i) and with (ii) selenite after 48h incubation times, respectively. Reduction of SeO_3^{2-}(10mg L^{-1}) to red Se^0 by *Mc. capsulatus* (Bath) (b) without (i) and with (ii) methane after 24 h incubation in both cases using the optimum temperature for the growth each bacterium.

S2a. Time course of selenite reduction after an initial addition of 10mg L^{-1} of the oxyanion to the culture medium containing *Mc. capsulatus* (Bath) (i) and *Ms trichosporium* OB3b (ii), respectively.

S2b. The effect on the time course of selenite reduction after an initial addition of 10, 40 and 100mg L^{-1} of the oxyanion to the culture medium containing *Mc. capsulatus* (Bath) (i), and after an initial addition of 10, 20 and 40mg L^{-1} of the oxyanion to the culture medium containing *Ms trichosporium* OB3b (ii), respectively.

S3. The variation of the mean selenate concentrations with time after incubation in the culture medium containing *Mc. capsulatus* (Bath) (a) and *Ms trichosporium* OB3b (b), respectively.

S4. The frequency distribution histogram of the selenium nanoparticles produced from the TEM images after the formation in the culture medium containing the methanotrophs *Mc. capsulatus* (Bath) (a) and *Ms trichosporium* OB3b (b), respectively.

S5. TEM micrographs of the cells and selenium nanoparticles at different incubation times 6, 24 and 48 h in the culture medium containing *Mc. capsulatus* (Bath)(i) and at incubation times of 48, 144 and 288 h in the culture medium containing *Ms trichosporium* OB3b(ii), respectively.

S6. Results of experiments showing selenite reduction after an initial addition of 100mg L^{-1} of the oxyanion incubated in the culture medium without methane in the presence of different cell factions: cell walls, cell membrane and cytoplasm of *Mc. capsulatus* (Bath) (a) and *Ms trichosporium* OB3b (b) immediately after the addition of the oxyanion and after72 h, respectively.
